# Psychoactive Substance Use and Its Association with Mental Health Symptomatology Among Latvian Medical Students: A Cross-Sectional Study

**DOI:** 10.3390/ijerph22121806

**Published:** 2025-11-30

**Authors:** Warnakulasuriya S. A. V. Fernando, Aviad David, Nicolo Cianci, Anastasija Sevcenko, Jelena Vrublevska, Elmars Rancans, Lubova Renemane

**Affiliations:** 1 Faculty of Medicine, Riga Stradiņš University, LV-1046 Riga, Latvia045161@rsu.edu.lv (N.C.);; 2 Department of Psychosomatic Medicine and Psychotherapy, Riga Stradiņš University, LV-1046 Riga, Latvia; 3 Department of Neuromedicine and Neurosciences, Faculty of Medicine and Life Sciences, University of Latvia, LV-1586 Riga, Latvia; 4 Department of Psychiatry and Narcology, Riga Stradiņš University, LV-1005 Riga, Latvia; elmars.rancans@rsu.lv (E.R.); lubova.renemane@rsu.lv (L.R.)

**Keywords:** psychoactive substances, medical students, resilience, anxiety, depression, intervention risk, mental health, medicine students, WHO ASSIST

## Abstract

Medical students are at elevated risk for psychoactive substance use and mental health challenges due to academic pressures and environmental stressors. This study aimed to determine the prevalence and trends of psychoactive substance use among medical students at Riga Stradins University (RSU) and to examine associations with symptoms of anxiety, depression, and resilience to stress. A bilingual, anonymous cross-sectional study was conducted using a SurveyMonkey-hosted questionnaire. The survey included a socio-demographic questionnaire, the Generalized Anxiety Disorder Questionnaire-7 (GAD-7), the Patient Health Questionnaire-9 (PHQ-9), the Brief Resilience Scale (BRS), and the World Health Organization Alcohol, Smoking, and Substance Involvement Screening Test (WHO ASSIST V3.1). A total of 559 RSU medical students participated (response rate: 31.8%). Lifetime substance use prevalence was highest for caffeine 98.7%, alcohol 93.9%, tobacco 68.4%, and cannabis 50.9%. High-risk use was noted for tobacco 6.8%, inhalants 4.2%, cocaine 3.6%, and alcohol 1.4%. Significant differences in total substance use were observed by gender (*p* = 0.006) and depression symptom severity by PHQ-9 (*p* < 0.001), which were predictors of the total involvement score. The findings suggest that further attention to mental health and substance use patterns among medical students may be beneficial for student well-being and professional development.

## 1. Introduction

Psychoactive substance use and associated attitudes among European medical students [1,2,3,4,5,6] and doctors in training are well documented [7]. The COVID-19 pandemic further intensified this concern, as a growing body of evidence indicates increased psychological distress among medical students [8,9,10]. Despite their medical knowledge, medical students are not immune to engaging in substance use. The demands of pre-clinical education, frequent exposure to human suffering during clinical training, and rigorous professional expectations throughout their careers contribute to elevated levels of psychological distress [11,12,13,14]. These factors may also erode resilience [15], which is broadly understood as the ability to bounce back or recover from stress [16]. 

A recent meta-analysis estimated anxiety symptom prevalence at 33.8% among medical students [13], compared with approximately 13% in the general European adult population before COVID-19 [17]. Similarly, depressive symptoms affect approximately 27.2–28.0% of medical students [12,14], far exceeding the 6.5% prevalence reported in the general European population [18]. Furthermore, inter-country variability has been documented both during and in the aftermath of the COVID-19 pandemic [18]. Although methodological differences such as sampling techniques persist, these symptom-based comparisons clearly highlight the disproportionate mental health burden among medical students.

Substance use has been documented as a maladaptive coping strategy among medical students and physicians in training [5,7,19,20]. A systematic review found that 24% of medical students reported risky or harmful alcohol use, with European populations at higher risk, and that 17.2% were current tobacco smokers [21]. Additional estimates included 11.8% past-month cannabis use, 9.9% past-month sedative use, and 7.7% stimulant use (mainly exam-related) [21]. Other psychoactive substances, such as cocaine, hallucinogens, and opioids (with the specific exception of Iran), generally remained below 3%. However, inhalant use varied regionally, with approximately 14.3% lifetime inhalant use concentrated in Latin America [21]. A more recent meta-analysis estimated lifetime cannabis use at 31.4% and past year use at 17.2%, with a singular European study reporting substantially higher rates [22].

Specific data on the use of stimulants, including for academic performance, is reported in 5.2–47.4% of medical students globally, with large regional variability [21,23,24]. Sedative and tranquilizer use also remains prevalent, with up to 25% of students reporting sedative use [21]. These trends reflect both recreational experimentation and maladaptive coping behaviors shaped by cultural and socioeconomic factors [25,26,27].

Latvia, a Baltic member state of the European Union (EU), provides internationally aligned and EU-accredited medical education programs within the Bologna framework [28]. Medical education is primarily delivered by Riga Stradins University (RSU) and the University of Latvia, both offering six-year Medical Doctor (M.D.) programs for domestic and international students, with admission being highly competitive and based on academic performance [28]. According to Eurostat data from the European Commission, Latvia ranks third among EU member states in the number of medical doctor graduates [29]. As such, RSU’s medical education program is internationally oriented and supported by global accreditation and certification frameworks [28,30].

Research on substance use trends among Latvian medical students is limited, particularly regarding associations with mental health outcomes across different psychoactive substances. These limitations have implications for student well-being and broader public health. Prior research conducted at RSU, while insightful and complementary to the present study, was constrained by a smaller sample size, selective recruitment, and a primary focus on mental health outcomes [31]. Addressing these knowledge gaps is particularly important, as today’s medical students represent potential future clinicians in a multitude of national and transnational healthcare systems. Consequently, the aim of the present study was to evaluate the relationships between psychoactive substance use, depressive and anxiety symptoms, resilience levels, and relevant sociodemographic factors in this population.

## 2. Materials and Methods

### 2.1. Study Design and Sampling

This observational, cross-sectional study was conducted among Latvian and international medical students in the Faculty of Medicine at RSU. A pilot study (n = 29) was carried out using convenience sampling via email invitation and included 14 international and 15 Latvian students from diverse academic disciplines. The pilot aimed to refine the questionnaire regarding clarity, length, usability, technical function, and data privacy based on structured feedback.

Data collection for the main study was conducted between June and October 2024 using SurveyMonkey, an online survey platform compliant with the General Data Protection Regulation (GDPR) and previously used in peer-reviewed healthcare research [32,33]. The final survey comprised a sociodemographic section and four standardized instruments: The World Health Organization Alcohol, Smoking, and Substance Involvement Screening Test (WHO ASSIST V3.1) for substance use, the Generalized Anxiety Disorder 7 (GAD-7) for anxiety symptoms, the Patient Health Questionnaire 9 (PHQ-9) for depressive symptoms, and the Brief Resilience Scale (BRS) for resilience assessment.

### 2.2. Inclusion and Exclusion Criteria

All students enrolled in the M.D. program at RSU who demonstrated sufficient proficiency in either Latvian or English were eligible to participate. The M.D. program is a 6-year course comprising 360 European Credit Transfer and Accumulation System (ECTS) credits, corresponding to level 7 of the European Qualifications Framework [28]. In instances where participants fulfilled the formal eligibility criteria but self-identified as non-eligible, their self-classification was respected, and these responses were excluded from the analytic sample.

### 2.3. Ethics and Ethical Considerations

This study was conducted in accordance with the ethical principles outlined in the Declaration of Helsinki for research involving human participants. Ethical approval was obtained from the RSU Research Ethics Committee in February 2024 (Document Nr. 2-PEK-4/163/2024). Participation was entirely voluntary and anonymous. Electronic informed consent was obtained prior to the initiation of the online questionnaire, and participants were informed that they could withdraw from the study at any time without penalty.

### 2.4. Measurement Tools and Procedures 

The WHO ASSIST V3.1, developed by the World Health Organization, is a standardized primary care screening instrument comprising eight items that assess both lifetime and recent (past three months) use of ten substance classes: tobacco products, alcohol, cannabis, cocaine, amphetamine-type stimulants (ATS), inhalants, sedatives and sleeping pills (e.g., benzodiazepines), hallucinogens, opioids, and other drugs [34,35]. For each substance, a substance-specific involvement (sum of items 2–7) classifies individuals into low, moderate, or high-risk categories, indicating the level of intervention required. An aggregate Total Involvement Score (TIS) can also be derived by summing across all substances. Although the TIS is not applied clinically, it has been used in research contexts [36]; in this study, it served as the dependent outcome in the regression analysis.

To capture additional psychoactive substances not included in the WHO ASSIST, a appendix (Table A1) was developed to assess lifetime (ever used) and past three-month use. The appendix was administered separately, following completion of the WHO ASSIST, to preserve the integrity of the validated instrument. The appendix included pharmacological classes with specified brand names to facilitate accurate reporting, particularly in relation to variations in legal status and accessibility across countries [37,38]. This appendix represented an exploratory adaptation specific to the current study and was not part of the validated WHO ASSIST instrument.

The appendix substances included caffeine, antidepressants, anxiolytics, and medical stimulants. Excluding caffeine, these represent prescription medications used in the treatment of psychiatric conditions. In this exploratory framework, anxiolytics and antidepressants were assessed, including selective serotonin reuptake inhibitors (SSRIs), serotonin antagonists and reuptake inhibitors (SARIs), and norepinephrine–dopamine reuptake inhibitors (NDRIs). SSRIs are established as first-line pharmacological treatments for anxiety disorders, whereas SARIs and NDRIs are primarily antidepressants with limited evidence for anxiolytic benefit, occasionally prescribed in patients with comorbid depression and anxiety [39]. Antidepressant use was assessed through self-reported use of SSRIs, SARIs, serotonin–norepinephrine reuptake inhibitors (SNRIs), and NDRIs, reflecting both therapeutic and potential off-label use [40,41], while anxiolytic use also included benzodiazepines and γ-aminobutyric acid (GABA) analogs [39]. Medical stimulant use refers to amphetamine and methylphenidate derivatives, which are increasingly reported among student populations [42,43,44]. Caffeine, the most widely consumed psychoactive substance globally [45,46], was included due to its emerging associations with psychiatric symptoms and comorbidities [47,48]. 

The BRS was used to assess participants’ ability to recover from stress, i.e., resilience [16]. The six-item BRS employs a five-point Likert scale with both positively and negatively worded items, reverse scored as appropriate. Higher mean scores (ranging from 1–5) indicate greater resilience. The six-item BRS has demonstrated good internal consistency and construct validity [16,49]. 

Depressive symptoms were assessed using the PHQ-9, a nine-item validated self-report screening instrument measuring depression symptom severity over the previous two weeks [50]. The PHQ-9 demonstrated good internal consistency and criterion validity [50]. The PHQ-9 assesses depression symptomatology on a scale of 0 to 27, where scores of 0 to 4 indicate minimal or no depression, 5 to 9: mild depression, 10 to 14: moderate depression, 15 to 19: moderately severe depression, and ≥20: severe depressive symptoms [50]. The presence of clinically relevant depressive symptoms was defined using a cut-off score of ≥10 [50]. 

Anxiety symptoms were assessed using the GAD-7, a seven-item validated self-report screening instrument evaluating generalized anxiety symptoms over the previous two weeks [51]. The GAD-7 assesses general anxiety symptomatology on a scale of 0 to 21, where scores of 0 to 4 indicate none to minimal anxiety, 5 to 9: mild anxiety, 10 to 14: moderate anxiety, and ≥15: severe anxiety symptoms [51]. The GAD-7 demonstrates high internal consistency, and the presence of clinically relevant anxiety symptoms was defined using a cut-off score of ≥10 [51].

To minimize language bias, participants completed questionnaires in their primary language of medical instruction (Latvian or English). 

Validated bilingual versions of the PHQ-9 [52] and GAD-7 [53], as well as the registered Latvian adaptation of the BRS [54], were used. The WHO ASSIST was translated into Latvian by an external bilingual contributor and back-translated into English in collaboration with the authors. This preliminary adaptation was developed for use in the present exploratory study. Sociodemographic data were collected to explore trends related to substance use, and participants were categorized as preclinical (Years 1–3) and clinical (Years 4–6). To standardize reporting of recent psychiatric treatment, a 90-day recall window (≈three months) was applied, reflecting a practical short-term observation period shown to reduce recall bias in healthcare surveys [55]. 

### 2.5. Data Collection Procedure 

The required minimum sample size was calculated using Cochran’s formula for sample size estimation based on a 95% confidence level (α = 0.05), resulting in a target sample size of n = 323. Survey invitations and the access link were distributed through the official RSU email system to all students enrolled in the M.D. program.

Additional recruitment was conducted electronically via social media channels, including RSU student associations, WhatsApp groups, Instagram, and semester-specific chat groups using a single survey link. To minimize ineligible or duplicate entries [32], safeguards included restricting submissions to one per IP address and requiring participants to manually enter their age as an open-ended response for verification.

### 2.6. Statistical Analysis

Data were screened for entry errors and typographical inconsistencies, and variables were recorded into categorical bins where appropriate. To ensure validity, only fully completed instruments and WHO ASSIST scores were retained for analysis. Missing data were handled using a combined approach. Listwise deletion was applied for all multivariable analyses, consistent with default procedures in IBM SPSS Statistics (version 29.0.2.0). Pairwise deletion was used for descriptive statistics and other bivariate analyses to maximize available data. Descriptive statistics and normality testing were conducted, followed by the application of parametric or nonparametric tests. All tests were two-tailed, with statistical significance set at α = 0.05. 

Continuous measures were summarized as mean ± standard deviation (M ± SD) when normally distributed, and as median with interquartile ranges [IQR] when non-normally distributed. Associations between categorical variables were examined using Pearson’s chi-squared test (*χ*^2^). Only variables meeting chi-square assumptions (minimum expected cell count ≥ 5) were retained, and results are reported as *χ*^2^ and corresponding *p*-values. Between-group differences in continuous nonparametric variables were assessed using the Mann–Whitney *U* test (two groups) or the Kruskal–Wallis test (more than two groups). For Mann–Whitney comparisons, Hodges–Lehmann estimators (HL) with 95% confidence intervals (CI) were provided as measures of effect size. When the Kruskal–Wallis test indicated significant omnibus differences, pairwise Mann–Whitney U tests with Bonferroni correction were applied. Parametric comparisons between two independent groups were performed using Welch’s *t* test, with results reported as *t*(*df*), *p*-values, and 95% confidence intervals for the mean difference (MD). 

Predictor variables identified in preliminary analyses and from existing literature were entered into a Generalized Linear Model (GLM). Given the over-dispersed, count-like distribution of the dependent variable (TIS), a negative binomial regression with a log link was applied. Multicollinearity among predictors was examined using Variance Inflation Factors (VIF) and tolerance statistics. Backward elimination, guided by statistical significance (*p* < 0.05), theoretical considerations, and relative model fit (Akaike’s Information Criterion, AIC), was used to derive a parsimonious final model.

## 3. Results

### 3.1. Socio-Demographic Characteristics of the Study Sample 

**Figure 1:**
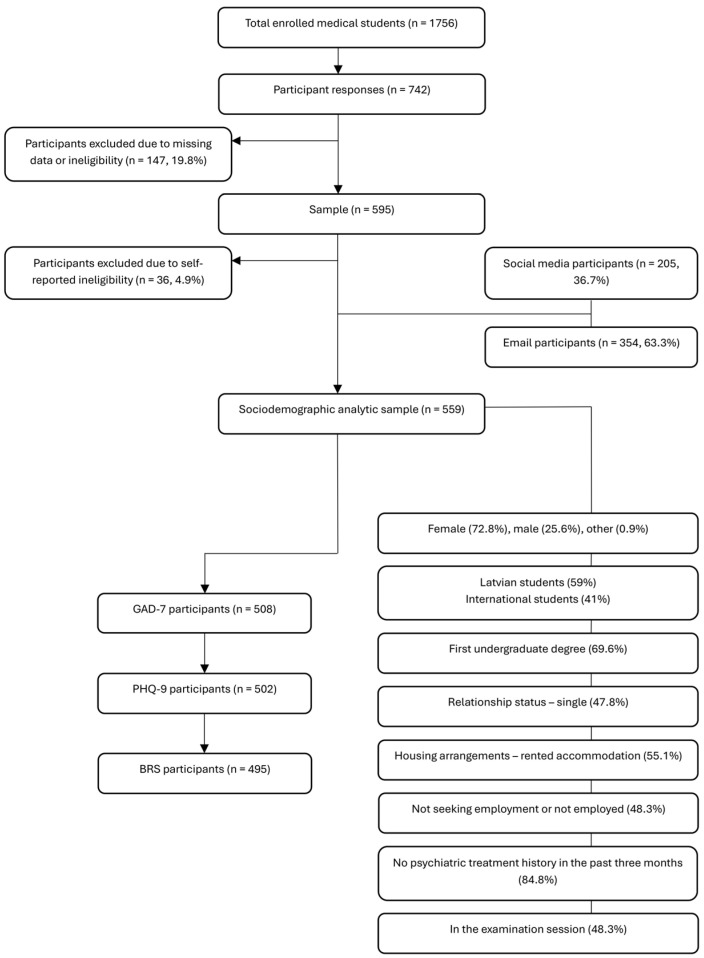
Participant flow diagram illustrating the recruitment, exclusion, and final analytic sample of medical students (n = 559), including subsamples completing the GAD-7, PHQ-9, and BRS.

Of the 1,756 medical students actively enrolled during the study period, approximately 31.8% completed the questionnaire (Figure 1). In total, 742 responses were collected. After excluding 183 ineligible or incomplete responses, the final analytic sample comprised 559 medical students. Participants were recruited primarily via RSU email (n = 354, 63.3%), and additionally through a social media link (n = 205, 36.7%).

Latvian students represented 59% of participants, while international students accounted for 41%. The sample was predominantly female (72.8%), followed by male (25.6%), and other gender identities (0.9%). The mean age was 22.3 years (SD = 4.1). Nearly half of the participants were first-year students (43.5%), and most were pursuing medicine as their first undergraduate degree (69.6%). Most participants reported having no children (92.7%), 47.8% were single, and 55.1% rented accommodation.

Approximately 48.3% of participants were not employed or seeking work. The most commonly reported combined monthly household income, representing the total income of all household members, excluding grants and loans, was between EUR 2001–EUR 5000 (24.2%). The majority (84.8%) had not accessed mental health care in the past three months. Among those with a treatment history, 10.4% had received psychotherapy alone, while 11.3% reported both psychotherapy and pharmacotherapy. 

Data collection was nearly evenly distributed between examination and non-examination periods (48.3% vs. 49.7%). Detailed socio-demographic characteristics of the study sample are presented in Table 1.

#### 3.1.1. Measurement Instruments (PHQ-9, GAD-7, BRS) and Their Trends 

The median scores for the total sample were 10.0 [5.0–15.0] for the GAD-7, and 9.0 [4.8–15.0] for the PHQ-9, while the mean BRS score was 3.25 ± 0.88. Overall, the prevalence of clinically relevant anxiety and depression symptoms (≥10) was 51.0% and 47.5%, respectively (Table 2). Female students reported higher anxiety and depression symptom scores than males. Median GAD-7 scores were 11.0 [7.0–15.0] in females versus 5.0 [3.0–11.0] in males (*U* = 14,495.50, HL = 4.0, 95% CI [3.0–6.0], *p* < 0.001). Similarly, median PHQ-9 scores were 10.0 [5.0–15.5] versus 6.0 [3.0–13.0], respectively (*U* = 17,429.50, HL = 3.0, 95% CI [2.0–4.0], *p* < 0.001). Gender differences were also evident in categorical symptom distributions for both anxiety (*χ*^2^(3) = 53.82, *p* < 0.001) and depression (*χ*^2^(4) = 31.23, *p* < 0.001). Females were more frequently represented above the clinically relevant symptom threshold for both anxiety and depression compared with males (58.8% vs. 27.7% and 53.2% vs. 32.3%, respectively).

Resilience scores were significantly lower among females (3.10 ± 0.82) than males (3.69 ± 0.92), *t*(201.9) = −6.34, *p* < 0.001, MD = −0.59 (95% CI [−0.77–−0.40]). Categorical analyses supported this difference (*χ*^2^(2) = 48.87, *p* < 0.001), with females more frequently classified as low-resilience (41.8% vs. 22.8%), while males predominated in the high-resilience group (33.1% vs. 8.3%).

Latvian-origin students reported higher anxiety and depression symptom scores compared with international students studying in Latvia. Median GAD-7 scores were 11.0 [7.0–15.0] among Latvian students and 7.0 [4.0–12.5] among international students (*U* = 39,130.00, HL = −3.0, 95% CI [−4.0–−2.0], *p* < 0.001; Table 3). Median PHQ-9 scores were also higher among Latvian students (11.0 [6.0–16.0]) compared with international students (7.0 [3.0–12.0]; *U* = 39,321.50, HL = −3.0, 95% CI [−5.0–−2.0], *p* < 0.001). Student origin differences were also evident in categorical symptom distributions for both anxiety (*χ*^2^(3) = 28.64, *p* < 0.001) and depression (*χ*^2^(4) = 38.41, *p* < 0.001). Latvian-origin students were more frequently represented above the clinically relevant symptom threshold for both anxiety and depression, compared with international students (57.9% vs. 41.2% and 56.7% vs. 34.9%, respectively). Resilience scores were significantly lower among Latvian origin students (3.03 ± 0.83) than international medical students (3.54 ± 0.87), *t*(432.2) = −6.45, *p* < 0.001, MD = −0.50 (95% CI [−0.66–−0.35]). Categorical analyses supported this difference (*χ*^2^(2) = 32.63, *p* < 0.001), with Latvian students more frequently classified as low resilience (45.6% vs. 26.0%), while international students were more often classified as high-resilience (23.6% vs. 8.1%).

#### 3.1.2. Sociodemographic Factors and PHQ-9 Scores

Pre-clinical students reported higher PHQ-9 scores compared to clinical students (HL = 2.0, 95% CI [1.0–3.0]; *U* = 22,695.00, *p* = 0.002; Table 4).

### 3.2. WHO ASSIST Substance Use Trends

#### Substance Use and WHO ASSIST Risk Grouping

The most commonly reported psychoactive substances were alcohol, tobacco, and cannabis. Lifetime substance use prevalence was highest for alcohol (93.9%), followed by tobacco (68.4%) and cannabis (50.9%). The corresponding past-three-month prevalence rates were 84.6%, 49.5%, and 19.8%, respectively (Table 5). Among supplementary substances, caffeine was most frequently reported (98.7% lifetime; 97.0% past three months), followed by antidepressants (16.8% lifetime; 8.9% past three months) and anxiolytics (14.7% lifetime; 7.9% past three months).

Gender comparisons revealed higher lifetime prevalence of tobacco (78.2% vs. 65.3%) and cannabis (62.4% vs. 47.4%) among males, while alcohol and caffeine use were comparable between genders. Therapeutic agent use was more frequent among females, including antidepressants (18.6% vs. 10.8%) and anxiolytics (15.3% vs. 13.8%). Less prevalent psychoactive substances included sedatives and amphetamine-type stimulants (13.9% each), cocaine (10.6%), hallucinogens (10.5%), inhalants (4.6%), opioids (2.5%), other drugs (3.4%), and any non-medical drugs by injection (0.6%) (Table 5).

**Figure 2:**
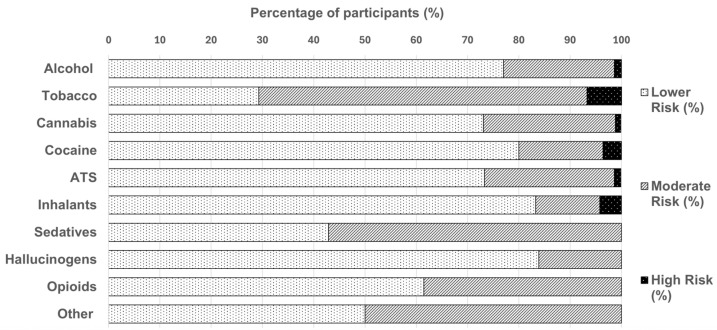
Proportional distribution of WHO ASSIST risk levels (lower, moderate, and high) for absolute psychoactive substance use in the total sample. WHO ASSIST risk classifications were obtained for alcohol (n = 495), tobacco (n = 369), cannabis (n = 268), cocaine (n = 55), ATS (amphetamine type-stimulants; n = 75), inhalants (n = 24), sedatives (n = 77), hallucinogens (n = 56), opioids (n = 13), and other substances (n = 20). WHO ASSIST = World Health Organization Alcohol, Smoking, and Substance Involvement Screening Test V3.1.

The WHO ASSIST substances associated with high-risk use were tobacco (6.8%), inhalants (4.2%), cocaine (3.6%), alcohol (1.4%), amphetamine-type stimulants (1.3%), and cannabis (1.1%) (Figure 2). Additionally, among those who reported use of other drugs (n = 19), 50% were classified as medium risk.

The negative binomial regression model was statistically significant compared with the intercept-only model (*χ*^2^(2) = 28.16, *p* < 0.001; Table 6). PHQ-9 scores were positively associated with the TIS, with each one-point increase on the PHQ-9 predicting a 3.4% increase in expected TIS (B = 0.033, SE = 0.007, 95% CI [0.019–0.046], Exp(B) = 1.034, *p* < 0.001). Gender also significantly predicted the TIS, with females having 26% lower expected scores than males (B = −0.299, SE = 0.108, 95% CI [−0.510–−0.088], Exp(B) = 0.741, *p* = 0.006). Model diagnostics indicated an adequate fit (Deviance/*df* = 1.04; AIC = 3731.33). In addition, when entered separately into adjusted negative binomial models, controlling for gender and PHQ-9 scores, student origin (Latvian vs. international; *p* = 0.054) and living arrangement (*p* = 0.063) demonstrated marginal significance. The omnibus test statistics for the preliminary negative binomial regression model are provided in Table A2, and the full predictor-level parameter estimates from that model are presented in Table A3.

### 3.3. Additional Findings and Miscellaneous Results 

Within the group of psychoactive substances classified as other in the appendix (n = 39), the most frequently reported classes were hallucinogens (35%), stimulants (25%), sedatives and anxiolytics (20%), nicotine products (10%), and antidepressants (10%). Open-ended responses recorded in the other category of the WHO ASSIST (n = 19) identified 32 distinct substances, reflecting multiple mentions per participant. The most reported were 2C-B (15.8%), 3,4-methylenedioxymethamphetamine (MDMA)/ecstasy (15.8%), and poppers/alkyl nitrites (10.5%).

## 4. Discussion

Women represented 70.8% of the faculty’s total student body. Consistent with this distribution, women were also strongly represented in our sample, comprising 72.8% of participants. This pattern reflects the documented increase in female participation in medical education across Europe [56]. Among international students, a majority originated from Western Europe (63.2%), reflecting cross-border enrollment trends within EU medical education. Regarding socioeconomic indicators, household income responses indicated a heterogeneous distribution; however, interpretation is limited by a 24.0% nonresponse rate. The rate of income nondisclosure in our sample aligns with European survey patterns, where approximately 20–40% of respondents in some countries decline to disclose household income [57]. Given the absence of more detailed socioeconomic measures (such as parental education and occupation), the reported income distribution appeared less skewed relative to comparable datasets. For example, data from Germany [58] and the United Kingdom [59,60] demonstrate that medical students are often overrepresented among higher-income families. 

Most students reported no prior history of psychiatric treatment; however, a notable minority (13.6%) reported having accessed treatment within the past three months. This finding is consistent with meta-analytic evidence indicating elevated levels of depression and anxiety symptoms among medical students [12,13,14]. Overall, our sample mirrors European trends of gendered mental health, which may have implications for female medical students in their specialty choice and training pathways at both the European [56,61] and local institutional level [28].

### 4.1. Substance Use Trends 

The lifetime prevalence of psychoactive substance use in our sample—particularly for alcohol (93.9%), tobacco (68.4%), and cannabis (50.9%)—was higher than the rates reported in a WHO ASSIST–based study of Swiss medical students (n = 886). That study reported lower lifetime rates of alcohol (86.6%), tobacco (31.4%), and cannabis use (23.5%) [4]. Specific data on regular use and dependence among medical schools in France and Italy indicate further heterogeneity across European medical schools. Pre-COVID-19 data from France (n = 171) reported higher regular alcohol (97%) and cannabis use (77%), but lower regular tobacco use (21%) [5]. In contrast, Italian data collected during the COVID-19 pandemic (n = 222) reported lower regular rates overall for alcohol (66%), cannabis use (6%), and tobacco use (25%) [62]. These differences may partly reflect national variations in psychoactive substance use, reporting methodology (lifetime vs. regular), or sampling methods.

Caffeine use was nearly universal among participants, exceeding reported rates of coffee consumption in comparable medical student samples. The lifetime prevalence of caffeine-containing substance use in our sample was 98.7%, surpassing reported rates of lifetime coffee consumption alone (n = 301, 82.9%) [63] and habitual coffee consumption (n = 583, 91.4%) [64] observed in two independent Italian samples of medical students. This difference likely reflects the broader definition of caffeine use in our study, which encompassed all caffeinated substances.

With respect to other substances, Swiss medical students reported lower lifetime prevalence for cocaine (1.4% vs. 10.6%), hallucinogens (1% vs. 10.5%), opioids (1.2% vs. 2.5%), and sedatives (6.8% vs. 13.9%) [4]. Recent use was also lower in the Swiss study, with only 3% reporting monthly sedative use and ≤0.3% reporting opioid and stimulant use. Overall, these findings support evidence that alcohol, tobacco, and cannabis remain the most commonly used substances among medical students across European contexts [21,22], while also highlighting considerable local variation.

Our study identified higher rates of lifetime and past-three-month prevalence of antidepressants and anxiolytics compared to other datasets (Table 5). In total, 10.4% of students reported receiving psychotherapy, 6.3% pharmacotherapy only, and 11.3% in combination with psychotherapy, while 28.7% reported any form of psychiatric treatment (Table 1). By contrast, a large French cross-sectional cohort of medical students (n = 10,985) reported lower rates of daily use, with 5.7% for anxiolytics and 2.8% for antidepressants, indicating that ongoing pharmacotherapy was less frequent in that context [65].

The observed gradient (regular use < recent use < lifetime use) may reflect both measurement differences and real-world factors such as intermittent adherence, misdiagnosis, other comorbidities, and substance use [39,40]. In support of this, substance use trends among medical students are possibly under-reported due to access, stigma, or variable approaches in treatment [66]. These factors may obscure actual patterns of pharmacotherapy uptake, even in the presence of clinical need [14]. 

Cognitive enhancement substance use also emerged as a relevant factor in understanding patterns of stimulant use among medical students. A Lithuanian study (n = 579) reported the use of nootropics, ATS, and related agents among 8.1% of medical students [67], while a large Belgian survey of university students (n = 12,144) reported lifetime medical stimulant use of 6.9% [43]. The corresponding lifetime prevalence in our sample was 9.5%, slightly higher than both the Lithuanian and Belgian figures, yet still within the ranges identified in international reviews [21,23,24]. This study also distinguished prescription stimulant use from non-prescription stimulant use, revealing a prevalence difference of approximately 4% (Table 5). However, stimulant use in medical students is primarily guided by psychiatric comorbidity [23,68], cognitive enhancement motives [21,23,24,67,68], and polysubstance use [38,68]. Therefore, potential confounding effects must be examined prior to more definitive conclusions. Furthermore, psychotropic medication use may also contribute to confounding within psychiatric screening outcomes and reported psychoactive substance use prevalence.

In summary, these findings suggest that psychotropic medication exposure is relatively common in this sample, with observed differences likely reflecting variability in treatment access, and treatment engagement shaped by both individual and systemic factors beyond the immediate medical school context [66,69]. Furthermore, our lifetime prevalence for psychoactive substances was broadly comparable with European studies, although differences in methodology and the limited availability of recent post-COVID-19 data among medical students constrain more definitive comparisons.

Distinct gendered patterns were observed: males reported higher use across all WHO ASSIST substances, whereas females reported greater use of antidepressants and anxiolytics (Table 5). These findings align with previous European research. At the Medical University of Vienna, a WHO ASSIST–based study of medical students (n = 589) found that males generally reported a higher past three-month prevalence of psychoactive substance use [70]. The only exception was sedatives, where females reported slightly higher rates in the 2nd (5.1% vs. 4.8%) and 6th years (2.8% vs. 0.0%) [70]. 

Although initial Mann–Whitney *U* and Kruskal–Wallis tests suggested differences in WHO ASSIST substance use risk scores by student origin and year of study (Figure 2), these effects were not confirmed when examining Hodges–Lehmann confidence intervals. This indicates that the apparent group differences may reflect statistical noise rather than meaningful variation.

Of particular relevance, our data also highlighted the use of 2C-B (a psychedelic phenethylamine with hallucinogenic and entactogenic properties) [71] and poppers (alkyl nitrites), substances often associated with chemsex and linked to adverse mental health outcomes [72]. A Belgian population study (n = 836) reported chemsex engagement among 30.9% of participants, most commonly with poppers (73%) and, to a lesser extent, 2C-B (3%) [73]. Similarly, a Parisian study of university students (n = 153) reported past-year hallucinogen (9.8%) and poppers use (5.2%), with chemsex behavior significantly associated with medical training (*p* = 0.021) [74]. Although limited in scope, these findings underscore shifting motivations for recreational polysubstance use, where psychoactive and physiological effects within distinct sociodemographic niches warrant further investigation.

### 4.2. Associations Between Demographics and Depression, Anxiety, and Resilience Scores

The proportion of students screening positive (≥10) for depression (PHQ-9) and anxiety (GAD-7) in our sample (Table 2 and Table 3) exceeded pooled global prevalence estimates reported in prior meta-analyses [12,13,14]. This difference may partly reflect significant variability by gender and student origin. Female students reported higher median scores on both the GAD-7 and the PHQ-9 compared with males (Table 2), a pattern consistent with global findings. For example, a multicenter study of 40 United States (U.S.) medical schools (n = 1428) conducted during the COVID-19 pandemic reported higher median scores among females than males (GAD-7: 7.0 vs. 5.0, *p* < 0.00001; PHQ-9: 6.0 vs. 4.0, *p* < 0.00001) [8]. Gender disparities in symptom burden have been associated with coping strategies such as self-distraction, behavioral disengagement, and denial [75]. These findings align with global data indicating a higher prevalence of depressive symptoms (31.5% vs. 24.2%) and an approximately two-fold greater likelihood of experiencing anxiety among female medical students [76]. However, gender-based differences are evident at a broader population level before [77,78] and during the COVID-19 pandemic [79]. In addition, the six-month timeframe of this study limits temporal comparability and precludes conclusions regarding persistent gender-specific trends. Thus, the gender disparities likely reflect broader gender linked mental health patterns rather than phenomena unique to medical education.

Latvian medical students reported comparable rates of depression, but markedly higher rates of anxiety compared with a Lithuanian sample of multidisciplinary students (n = 1368; depression: 56.7% vs. 45.0%; anxiety: 57.9% vs. 38.0%) [80]. Prevalence estimates for Latvian medical students also exceeded those reported for European medical students during the COVID-19 pandemic, where pooled estimates of moderate-to-severe symptoms were 23.9% for depression (95% CI [18.1–29.8]) and 29.7% for anxiety (95% CI [13.2–46.2]) across five studies [9].

In contrast, international medical students in Latvia reported lower symptom levels (depression: 34.9%; anxiety: 41.2%), although these values still surpassed comparable European values. However, across broader European subgroups, overall symptom prevalence was higher (depression: 39.9%; anxiety: 45.0%), reflecting the inclusion of milder symptomatology [9], whereas the present study defined prevalence at moderate or greater symptom severity. Historical data from Estonian medical students (n = 413) assessed using the Emotional State Questionnaire identified anxiety symptoms in 21.9% and depressive symptoms in 30.6% of participants [81]. Although based on a pre-COVID-19 sample and a different instrument, these findings, together with data on Latvian origin students [31], suggest that elevated symptom prevalence within Baltic student populations (Latvian, Lithuanian, and Estonian) is not exclusive to the COVID-19 pandemic. Furthermore, potential variations in optimal cutoff thresholds and instrument sensitivities across psychiatric screening instruments may also contribute to higher apparent prevalence rates in the Baltic region [52,80].

Resilience differed significantly by gender and student origin (Table 2 and Table 3), although mean scores for all groups remained within the normal range. Comparable mean scores and gender-based patterns have been reported in other European studies conducted during the COVID-19 pandemic. A Serbian study reported a mean BRS score of 3.17 ± 0.80 among medical students, while 33.3% of all study participants were classified as low, 56.7% as medium, and 10% as high resilience [82]. Similarly, a Swedish study of medical students (n = 457; semesters 2–10) found lower resilience scores among females (*p* < 0.001), with most participants classified as normal resilience (53.1%), followed by low (27.9%) and high (19.0%) [83]. However, post hoc analysis did not confirm specific differences between groups, and the exclusion of early and final year students may limit direct comparability with our findings. Nevertheless, resilience levels observed in this study appear consistent with broader European patterns, despite gender and sample composition differences. 

Normality diagnostics indicated borderline to significant deviations across all psychiatric screening tools. Skewness Z-scores were 0.52 for the BRS, 1.43 for the GAD-7, and 4.98 for the PHQ-9, while kurtosis Z-scores ranged from 2.78 to 4.17. These values exceed accepted thresholds of normality (Z > 1.96), reflecting distributional properties typical of psychiatric screening instruments [84]. 

### 4.3. Year-of-Study Differences in Depression, Anxiety, and Resilience

The PHQ-9 results from this study align with large multicenter investigations that have demonstrated significant stage of study differences in depressive symptoms among medical students [1,8], as well as with meta-analytic evidence showing greater symptom burden among pre-clinical students [14]. However, other reviews, such as that by Rotenstein et al. [12], reported no significant stage-related differences, emphasizing sample heterogeneity and methodological limitations. In contrast, meta-analytic data have generally shown no significant stage differences for anxiety [13], which is consistent with the absence of stage effects in our GAD-7 data. Similarly, resilience may stabilize as students develop coping strategies or self-select out of medical training, resulting in comparable scores across study stages [83]. 

### 4.4. Associations Between Mental Health, Socio-Demographics, and Substance Use Risk

Our negative binomial regression model identified depressive symptoms and gender as significant predictors of substance involvement, as measured by the WHO TIS. Each additional PHQ-9 point corresponded to a 3.4% higher expected TIS, while female students had a 26% lower expected TIS compared with males (Table 6). These findings align with prior research among Polish [3] and Italian medical students [62], where male gender was linked to hazardous alcohol use. Similarly, data from Ethiopia indicate that gender and the interaction of gender with residence are significant predictors of risky substance use in a WHO ASSIST-based study on engineering students (n = 243) [85]. A comparable association was observed among U.S. registered nurses (n = 1478), where WHO ASSIST data linked depressive symptoms to increased substance use risk [86].

Furthermore, a Spanish study of medical students (n = 1265) reported higher depression symptom levels among females and gender differences in substance use patterns [1]. Several variables previously identified as predictors were not significant in our model. For example, age, study year, anxiety, and impulsiveness have been identified as predictors of substance use in a large multidisciplinary logistic regression study from a German University sample (n = 3991) [87]. Similarly, anxiety was a significant predictor across multivariate regression models examining substance use among registered nurses [86]. However, none of these variables predicted substance use involvement in our data, and housing arrangements only approached significance. 

These discrepancies likely reflect both contextual differences and the application of a parsimonious negative binomial model, which optimized explanatory power while addressing over-dispersion in count data (skewness = 2.16; kurtosis = 8.68). Nonetheless, variables excluded from our model may remain important in studies employing alternative analytic frameworks, such as logistic or linear regression.

### 4.5. Implications and Recommendations

The findings of this research highlight the importance of education, monitoring, and referral programs, which can be shaped by local context and supported according to institutional capacity. 

The Association of American Medical Colleges Guidelines for the Development of Chemical Impairment Policies for Medical Schools emphasize that chemical dependency is a treatable condition [88,89]. Institutional policies should therefore ensure confidentiality, encourage early self-referral, and support recovery while maintaining patient safety [88,89]. At RSU, existing policies prohibit the use of illegal psychoactive substances on University or affiliated clinical premises. In addition, smoking and vaping bans were introduced across RSU premises in October 2022. 

Future institutional policies should further emphasize stigma reduction surrounding psychoactive substance use, recognizing education as central to effective prevention [88,89,90]. Furthermore, brief mandatory modules focused on addiction medicine and harm-reduction principles may contribute to improved understanding, reduced stigma, greater empathetic awareness, and strengthened professional responsibility [89,90,91]. Incorporating these modules early and within the educational curriculum promotes and empowers a culture of both individual, collective, and institutional action [89,90,91]. This educational approach reframes substance use from a stigmatized disciplinary issue to a personal and public-health issue that requires evidence-based screening, early intervention, and empathetic, pragmatic support.

Effective policy depends on accurate, recent, and actionable data. A structured, longitudinal monitoring program is warranted to facilitate early detection and intervention. Institutional monitoring of both incoming and current medical students may help identify knowledge gaps, attitudes, and potentially problematic psychoactive substance use [4,91]. Furthermore, large cross-sectional datasets, including this study, consistently demonstrate an association between psychoactive substance use and psychological distress among medical students [1,4,92] and in the general population [93]. Therefore, integrated, time-sensitive monitoring of both psychoactive substance use and mental health is essential in medical education. 

Evidence-based screening tools such as the Brief Alcohol Screening and Intervention for College Students have proven effective in reducing alcohol related harms in university populations [94]. A pragmatic institutional solution would include an annual, anonymous questionnaire distributed to all RSU medical students to assess and identify at-risk students, monitor mental health, and identify students requiring a follow-up within a longer-term project such as the ETMED-L project [4]. The instruments used in this study demonstrate the value of anonymous bilingually validated tools for screening purposes: the WHO ASSIST V3.1, PHQ-9, GAD-7, and BRS. However, broader implementation of the WHO ASSIST remains contingent upon its successful validation in the Latvian language, with V3.0 recommended for online research applications [35]. 

Where risk or impairment is evident, referral to cognitive-behavioral therapy or motivational interviewing is recommended, ensuring confidentiality and clear criteria for return to training [4,88,89]. RSU offers an established support system for students. The RSU Health Centre provides medical and psychiatric care, while the Career Guidance and Well-Being Centre offers confidential counselling, crisis support, and self-referral pathways [95]. Additional mental health services are available through the Clinic of Psychosomatic Medicine and Psychotherapy. Recommended interventions can be flexibly integrated within this support framework to ensure timely and student-centered care. 

Integrating detection, harm reduction, and treatment pathways into a unified student-centered strategy fulfills ethical mandates while mitigating long-term professional risk. Moreover, this model ensures that RSU remains aligned with its mandate to safeguard medical student health, patient safety, and institutional integrity through confidentiality, support, and rehabilitation.

## 5. Limitations

Several limitations should be considered when interpreting these findings.

First, among all respondents (n = 742), 4.9% (n = 36) self-reported their ineligibility during survey completion. This self-reporting highlights concerns regarding perceived anonymity and privacy, particularly related to the disclosure of sensitive mental health and substance use information. As the study design prioritized anonymity and data quality, no participant contact details or identifying information were stored. Thus, participants did not receive individualized referral links or feedback related to their reported mental health or psychoactive substance use. However, future studies should more explicitly emphasize data protection and confidentiality while integrating self-referral information to university support services. Adopting a hybrid anonymous-confidential design could enhance participant well-being and trust in data protection procedures.

Second, the cross-sectional study design precludes causal inference. Although significant associations were observed, these reflect correlations at a single time point within a six-month data collection window; therefore, definitive conclusions on directionality are limited. More broadly, online survey-based research is vulnerable to convenience sampling, self-selection, and nonresponse biases. These factors may partly explain the significant deviations from normality observed in the continuous scores of the GAD-7, the PHQ-9, and the BRS. Such deviations may have reduced measurement sensitivity.

Third, approximately 70–80% of participants who reported substances under the other or appendix categories did so despite the WHO ASSIST providing comprehensive, predefined substance classifications. This pattern suggests ambiguity regarding substance categorization or a possible misinterpretation of survey items, potentially reflecting the preliminary nature of the Latvian adaptation of the WHO ASSIST. Future applications of the WHO ASSIST in Latvia should be contingent on completing the designated WHO validation process to ensure linguistic and conceptual equivalence. 

Fourth, the psychiatric screening tools (GAD-7, PHQ-9, BRS) assess symptoms over the preceding two weeks, whereas the WHO ASSIST evaluated substance use over the past three months. Although this temporal mismatch may introduce some asynchrony, it should be noted that the negative binomial regression model did not directly compare these measures but modeled their associations within the same analytic framework. Finally, the sample included medical students from only one of the two Latvian universities offering medical education, which may limit the generalizability. Although the sample was large and diverse, inclusion of medical students from the University of Latvia in future research would enhance representativeness.

Consequently, future investigations should refine methodological items and variables approaching significance in this study, while expanding the range of contextual, cultural, social, and religious factors assessed. Doing so may improve model validity and provide a more nuanced understanding of substance use behaviors among medical students in Latvia.

## 6. Conclusions

This study contributes novel data on substance use patterns, gender-specific differences, and mental health correlates among medical students in Latvia following the COVID-19 pandemic. The findings highlight the importance of targeted prevention strategies, enhanced education on substance use, and efforts to reduce stigma surrounding mental health support within medical training. Elevated prescription substance use and relatively high levels of recent (past-three-month) psychoactive substance consumption in male students, combined with a substantial proportion of female students exceeding standard clinical cutoffs (≥10) on the GAD-7 and the PHQ-9, warrant further investigation. Future longitudinal research should clarify causal relationships and incorporate sociodemographic and cultural factors that may shape both substance use and mental health trajectories in this population.

## Figures and Tables

**Table 1:** Sociodemographic profile of medical student participants (n = 559).

Variables	Categories	M ± SD
Mean age		22.3 ± 4.1
		**n (%)**
Gender	Male	143 (25.6)
	Female	407 (72.8)
	Other	5 (0.9)
	No response	4 (0.7)
Student origin	International students	229 (41.0)
	Latvian students	330 (59.0)
International student nationality *	German	64 (27.9)
	Swedish	36 (15.7)
	Finnish	23 (10.0)
	Italian	22 (9.6)
	Russian	22 (9.6)
	All other nationalities	62 (27.0)
Academic year	1	243 (43.5)
	2	60 (10.7)
	3	72 (12.9)
	4	47 (8.4)
	5	81 (14.5)
	6	49 (8.8)
	No response	7 (1.3)
Prior education	Attending university but no degree	389 (69.6)
	Associate’s degree	19 (3.4)
	Bachelor’s degree	47 (8.4)
	Graduate degree	69 (12.3)
	No response	35 (6.3)
Marital status	Single	267 (47.8)
	In a committed relationship	250 (44.7)
	Married	26 (4.7)
	Separated or widowed	12 (2.1)
	No response	4 (0.7)
Housing arrangements	Owned	58 (10.4)
	Rented	308 (55.1)
	Live with family or partner	186 (33.3)
	No response	7 (1.3)
Employment status	Employed > 160 h—full-time	29 (5.2)
	Employed < 160 h—part-time	185 (33.1)
	Not employed, looking for work	65 (11.6)
	Not employed, not looking for work	270 (48.3)
	No response	10 (1.8)
Any applicable psychiatric treatment	Pharmacotherapy	35 (6.3)
	Psychotherapy	58 (10.4)
	Psychotherapy and pharmacotherapy	63 (11.3)
	Brain stimulation therapy	1 (0.2)
	All other combinations	3 (0.5)
	None	384 (68.7)
	No response	15 (2.7)
Psychiatric treatment in the last 90 days	Yes	76 (13.6)
	No	474 (84.8)
	No response	9 (1.6)
Children	None	518 (92.7)
	One or More	33 (5.9)
	No response	8 (1.4)
Examination status	In the exam session	270 (48.3)
	Not in the exam session	278 (49.7)
	No response	11 (2.0)
Total monthly income earned by all members of the household, excluding grants and loans	EUR 0–1000	105 (18.8)
	EUR 1001–2000	88 (15.7)
	EUR 2001–5000	135 (24.2)
	EUR 5001–10,000	45 (8.1)
	EUR 10,001–20,000	25 (4.5)
	EUR 20,001 or more	27 (4.8)
	I do not wish to answer	127 (22.7)
	No response	7 (1.3)

Note: Continuous variables are presented as the mean ± standard deviation (M ± SD). Categorical variables are presented as n (%). Percentages may not total 100% due to rounding. * Subcategory calculated from n = 229.

**Table 2:** Gender differences in anxiety and depression symptoms and resilience scores among participants.

Instrument	Total Sample	Female	Male	Test Statistic	*p*
Continuous scores					
BRS	3.25 ± 0.88 (n = 495)	3.10 ± 0.82 (n = 361)	3.69 ± 0.92 (n = 127)	*t*(201.9) = −6.34; MD = −0.59, 95% CI [−0.77–−0.40]	*p* < 0.001
GAD-7	10.0 [5.0–15.0] (n = 508)	11.0 [7.0–15.0] (n = 371)	5.0 [3.0–11.0] (n = 130)	*U* = 14,495.50; HL = 4.0, 95% CI [3.0–6.0]	*p* < 0.001
PHQ-9	9.0 [4.8–15.0] (n = 502)	10.0 [5.0–15.5] (n = 365)	6.0 [3.0–13.0] (n = 130)	*U* = 17,429.50; HL = 3.0, 95% CI [2.0–4.0]	*p* < 0.001
Categorical classifications					
BRS categories	(n = 495)	(n = 361)	(n = 127)		
Low resilience (1.00–2.99)	185 (37.4%)	151 (41.8%)	29 (22.8%)	*χ*^2^(2) = 48.87	*p* < 0.001
Normal resilience (3.00–4.30)	238 (48.1%)	180 (49.9%)	56 (44.1%)		
High resilience (4.31–5.00)	72 (14.5%)	30 (8.3%)	42 (33.1%)		
GAD-7 Scores	(n = 508)	(n = 371)	(n = 130)		
None to minimal (0–4)	113 (22.2%)	56 (15.1%)	57 (43.8%)	*χ*^2^(3) = 53.82	*p* < 0.001
Mild (5–9)	136 (26.8%)	97 (26.1%)	37 (28.5%)		
Moderate (10–14)	124 (24.4%)	106 (28.6%)	17 (13.1%)		
Severe (15–21)	135 (26.6%)	112 (30.2%)	19 (14.6%)		
PHQ-9 Scores	(n = 502)	(n = 365)	(n = 130)		
None or minimal (0–4)	125 (24.9%)	69 (18.9%)	55 (42.3%)	*χ*^2^(4) = 31.23	*p* < 0.001
Mild (5–9)	138 (27.5%)	102 (27.9%)	33 (25.4%)		
Moderate (10–14)	110 (21.9%)	93 (25.5%)	17 (13.1%)		
Moderately severe (15–19)	63 (12.5%)	51 (14.0%)	10 (7.7%)		
Severe (20–27)	66 (13.1%)	50 (13.7%)	15 (11.5%)		

Note: Data are presented as the mean ± SD, median [IQR], or n (%). Denominators vary due to item-level missing responses (pairwise deletion), and the total sample includes participants with nonbinary or unspecified gender; therefore, subgroup counts may not sum to the total sample. Percentages may not total 100% due to rounding. GAD-7 = Generalized Anxiety Disorder-7; PHQ-9 = Patient Health Questionnaire-9; BRS = Brief Resilience Scale. All *p* < 0.05.

**Table 3:** Differences in anxiety, depression symptoms, and resilience scores between international and Latvian-origin medical students.

Instrument	Total Sample	International Students	Latvian Students	Test Statistic	*p*
Continuous scores					
BRS	3.25 ± 0.88 (n = 495)	3.54 ± 0.87 (n = 208)	3.03 ± 0.83 (n = 285)	*t*(432.2) = −6.45; MD = −0.50, 95% CI [−0.66–−0.35]	*p* < 0.001
GAD-7	10.0 [5.0–15.0] (n = 508)	7.0 [4.0–12.5] (n = 211)	11.0 [7.0–15.0] (n = 295)	*U* = 39,130.00; HL = −3.0, 95% CI [−4.0–−2.0]	*p* < 0.001
PHQ-9	9.0 [4.8–15.0] (n = 502)	7.0 [3.0–12.0] (n = 209)	11.0 [6.0–16.0] (n = 291)	*U* = 39,321.50; HL = −3.0, 95% CI [−5.0–−2.0]	*p* < 0.001
Categorical classifications					
BRS categories	(n = 495)	(n = 208)	(n = 285)		
Low resilience (1.00–2.99)	185 (37.4%)	54 (26.0%)	130 (45.6%)	*χ*^2^(2) = 32.63	*p* < 0.001
Normal resilience (3.00–4.30)	238 (48.1%)	105 (50.5%)	132 (46.3%)		
High resilience (4.31–5.00)	72 (14.5%)	49 (23.6%)	23 (8.1%)		
GAD-7 Scores	(n = 508)	(n = 211)	(n = 295)		
None to minimal (0–4)	113 (22.2%)	71 (33.6%)	42 (14.2%)	*χ*^2^(3) = 28.64	*p* < 0.001
Mild (5–9)	136 (26.8%)	53 (25.1%)	82 (27.8%)		
Moderate (10–14)	124 (24.4%)	45 (21.3%)	78 (26.4%)		
Severe (15–21)	135 (26.6%)	42 (19.9%)	93 (31.5%)		
PHQ-9 Scores	(n = 502)	(n = 209)	(n = 291)		
None or minimal (0–4)	125 (24.9%)	80 (38.3%)	45 (15.5%)	*χ*^2^(4) = 38.41	*p* < 0.001
Mild (5–9)	138 (27.5%)	56 (26.8%)	81 (27.8%)		
Moderate (10–14)	110 (21.9%)	37 (17.7%)	72 (24.7%)		
Moderately severe (15–19)	63 (12.5%)	17 (8.1%)	46 (15.8%)		
Severe (20–27)	66 (13.1%)	19 (9.1%)	47 (16.2%)		

Note: Data are presented as mean ± SD, median [IQR], or n (%). Denominators vary due to item-level missing responses (pairwise deletion), and the total sample includes participants who did not specify student origin; therefore, subgroup counts may not sum to the total sample. Percentages may not total 100% due to rounding. GAD-7 = Generalized Anxiety Disorder-7; PHQ-9 = Patient Health Questionnaire-9; BRS = Brief Resilience Scale. All *p* < 0.05.

**Table 4:** Group differences in PHQ-9 scores by year of study (n = 498; pre-clinical n = 333, clinical n = 165).

Dependent Variable	Independent Variable	Median [IQR]	Test Statistic	*p*
PHQ-9 total score	Clinical year	Pre-clinical: 10.0 [5.0–15.0] Clinical: 7.0 [3.0–14.0]	*U* = 22,695.00, HL = 2.0, 95% CI [1.0–3.0]	0.002

Note: *U* = Mann–Whitney *U* test with Hodges–Lehmann estimator and 95% CI. HL = Hodges–Lehmann estimator; CI = confidence interval. Group values are presented as median [IQR]. PHQ-9 = Patient Health Questionnaire-9. All *p* < 0.05.

**Table 5:** Lifetime substance use prevalence, past-three-month substance use prevalence, and gender differences for psychoactive substance use.

WHO ASSIST Psychoactive Substance	Lifetime Substance Use Prevalence, n (%)	Past-Three-Month Substance Use Prevalence, n (%)	Male Lifetime Substance Use Prevalence, n (%)	Female Lifetime Substance Use Prevalence, n (%)
Alcohol	505 (93.9%)	455 (84.6%)	131 (95.6%)	367 (93.1%)
Tobacco	379 (68.4%)	274 (49.5%)	111 (78.2%)	264 (65.3%)
Cannabis	270 (50.9%)	105 (19.8%)	83 (62.4%)	185 (47.4%)
Cocaine	56 (10.6%)	19 (3.6%)	23 (17.6%)	32 (8.2%)
Amphetamine-type stimulants	73 (13.9%)	25 (4.8%)	34 (26.2%)	39 (10.1%)
Inhalants	24 (4.6%)	4 (0.8%)	14 (10.8%)	9 (2.3%)
Sedatives	73 (13.9%)	49 (9.3%)	19 (14.6%)	54 (13.9%)
Hallucinogens	55 (10.5%)	23 (4.4%)	26 (20.0%)	29 (7.5%)
Opioids	13 (2.5%)	9 (1.7%)	6 (4.6%)	7 (1.8%)
Other	18 (3.4%)	12 (2.3%)	9 (6.9%)	9 (2.3%)
Any non-medical drug by injection	3 (0.6%)			
Supplementary psychoactive substances				
Caffeine	518 (98.7%)	509 (97.0%)	128 (98.5%)	383 (98.7%)
Antidepressants	87 (16.8%)	46 (8.9%)	14 (10.8%)	71 (18.6%)
Anxiolytics	76 (14.7%)	41 (7.9%)	18 (13.8%)	58 (15.3%)
Medical stimulants	49 (9.5%)	34 (6.6%)	22 (16.9%)	27 (7.1%)
Other	21 (4.1%)	14 (2.7%)	5 (3.8%)	16 (4.2%)

Note: Denominators vary across substances due to item-level missing responses. Percentages reflect item-level response counts. WHO ASSIST = World Health Organization Alcohol, Smoking, and Substance Involvement Screening Test V3.1.

**Table 6:** Negative binomial regression predicting the Total Involvement Score (TIS) from gender and PHQ-9 depression scores (n = 470).

Predictor	B	SE	95% CI for B	Wald *χ*^2^	*p*	Exp(B)
Intercept	2.821	0.112	[2.602–3.041]	636.704	<0.001	16.8
Gender (female vs. male)	−0.299	0.108	[−0.510–0.088]	7.692	0.006	0.741
PHQ-9 total score	0.033	0.007	[0.019–0.046]	21.713	<0.001	1.034

Note: Values are regression coefficients (B), standard errors (SE), 95% confidence intervals (CI), with Wald *χ*^2^ statistics, *p*-values, and exponentiated coefficients Exp(B). PHQ-9 = Patient Health Questionnaire-9. All *p* < 0.05.

## Data Availability

Due to privacy and ethical restrictions, the data generated and/or analyzed during the current study are not publicly available. De-identified, aggregate data may be made available upon reasonable request to the corresponding author, subject to approval by the institutional ethics committee and in compliance with applicable data protection regulations.

## Abbreviations

The following abbreviations are used in this manuscript:

WHO ASSIST	World Health Organization Alcohol, Smoking and Substance Involvement Screening Test
GAD-7	The Generalized Anxiety Disorder 7
PHQ-9	The Patient Health Questionnaire 9
BRS	The Brief Resilience Scale

## Appendix A

### Appendix A.1

**Table A1:** Lifetime substance use prevalence, and past-three-month substance use prevalence for supplementary psychoactive substance use.

**Question 1: In Your Life, Which of the Following Substances Have You Ever Used?**					
	Examples (pharmacological/brand names as in the original)			No	Yes
Caffeine	Coffee, tea, energy drinks, etc.			☐	☐
**Question 1.1: Please answer the following substances, if you have used them as medicine prescribed by a doctor/doctor’s prescription.**					
Antidepressants	Fluoxetine/Prozac, Paroxetine/Paxil, Sertraline/Zoloft, Escitalopram/Cipralex/Lexapro, Trazadone/Desyrel/Oleptro, Bupropion/Wellbutrin/Zyban, etc.			☐	☐
Anxiolytics	Alprazolam/Xanax/Niravam, Gabazolamine, Diazepam/Valium, Lorazepam/Ativan, etc.			☐	☐
Medicinal stimulants	Amphetamine/Dextroamphetamine/Adderall, Methylphenidate/Concerta,/Ritalin, etc.			☐	☐
Other (specify):				☐	☐
**Question 2: In the past three months, how often have you used the substances you mentioned (first drug, second drug, etc.)?**					
Category	Never	Once or twice	Monthly	Weekly	Daily or almost daily
Caffeine (coffee, tea, energy drinks, etc.)	0	2	3	4	6
Antidepressants (Fluoxetine/Prozac, Paroxetine/Paxil, Sertraline/Zoloft, Escitalopram/Cipralex/Lexapro, Trazadone/Desyrel/Oleptro, Bupropion/Wellbutrin/Zyban, etc.)	0	2	3	4	6
Anxiolytics (Alprazolam/Xanax/Niravam, Gabazolamine, Diazepam/Valium, Lorazepam/Ativan, etc.)	0	2	3	4	6
Medicinal stimulants (Amphetamine/Dextroamphetamine/Adderall, Methylphenidate/Concerta/Ritalin, etc.)	0	2	3	4	6
Other (specify):	0	2	3	4	6

### Appendix A.2

**Table A2:** Omnibus Wald *χ*^2^ tests from a preliminary generalized linear model (negative binomial regression) predicting the TIS (n = 466).

Predictor	Wald *χ*^2^	*df*	*p*
Intercept	572.458	1	<0.001
Gender	10.468	1	0.001
Living quarters	5.099	2	0.078
Student origin	3.085	1	0.079
PHQ-9 total score	3.458	1	0.063
GAD-7 total score	2.149	1	0.143
Clinical year	0.316	1	0.574

Note: Wald *χ*^2^ statistics, degrees of freedom (*df*), and corresponding *p*-values are presented. GAD-7 = Generalized Anxiety Disorder-7; PHQ-9 = Patient Health Questionnaire-9. All *p* < 0.05.

**Table A3:** Parameter estimates from a preliminary generalized linear model (negative binomial regression) predicting the TIS (n = 466).

Predictor	B	SE	95% CI for B	Wald *χ*^2^	*p*	Exp(B)
Intercept	2.692	0.165	[2.369–3.015]	266.545	<0.001	14.77
Gender (female vs. male)	−0.394	0.122	[−0.633–−0.155]	10.468	0.001	0.674
Living quarters (owned vs. family)	0.140	0.170	[−0.194–0.473]	0.676	0.411	1.150
Living quarters (rented vs. family)	0.310	0.137	[0.041–0.579]	5.099	0.024	1.364
Student origin (international vs. Latvian)	−0.241	0.137	[−0.510–0.028]	3.085	0.079	0.786
PHQ-9 total score	0.020	0.016	[−0.001–0.041]	3.458	0.063	1.020
GAD-7 total score	0.019	0.013	[−0.006–0.044]	2.149	0.143	1.019
Clinical year (pre-clinical vs. clinical)	0.061	0.108	[−0.152–0.274]	0.316	0.574	1.063

Note: Values are regression coefficients (B), standard errors (SE), 95% confidence intervals (CI), Wald *χ*^2^ statistics, exponentiated coefficients Exp(B) and corresponding *p*-values are presented. GAD-7 = Generalized Anxiety Disorder-7; PHQ-9 = Patient Health Questionnaire-9. All *p* < 0.05.
